# Cognitive appraisal, negative coping, and family resilience among acute leukemia patients and their primary family caregivers: an actor-partner interdependence mediation model of quality of life

**DOI:** 10.3389/fpsyg.2026.1842350

**Published:** 2026-09-18

**Authors:** Hezi Mu, Changchang Chen, Jinquan Li, Yi Cui, Xia Chen, Yinling Zhang, Weihong Li

**Affiliations:** 1 School of Nursing, Beijing University of Chinese Medicine, Beijing, China; 2 Department of Nursing, Air Force Medical University, Xi'an, China; 3 Nursing Department, Peking University Shenzhen Hospital, Shenzhen, China

**Keywords:** acute leukemia, cognitive appraisal, coping style, family resilience, primary family caregivers, quality of life

## Abstract

**Objectives:**

This study used a dyadic approach to examine the association between cognitive appraisal and quality of life (QoL), and to test whether negative coping and family resilience mediate this association in dyads of acute leukemia (AL) patients and their primary family caregivers.

**Methods:**

Three hundred and eight AL patient-caregiver dyads were recruited from five hospitals in China. QoL was measured using the Functional Assessment of Cancer Therapy-Leukemia (FACT-Leu) for patients and the 12-Item Short-Form Health Survey (SF-12) for primary family caregivers. Cognitive appraisal, negative coping, and family resilience were assessed using the Cognitive Appraisal of Health Scale (CAHS), the Trait Coping Style Questionnaire (TCSQ), and the Family Hardiness Index (FHI), respectively. The Actor-Partner Interdependence Mediation Model (APIMeM) was performed in R to examine direct and indirect effects.

**Results:**

Patients’ QoL was significantly correlated with their own positive coping (*r* = 0.523, *p* < 0.01), family resilience (*r* = 0.489, *p* < 0.01), and negative coping (*r* = −0.466, *p* < 0.01), and with caregivers’ QoL (*r* = 0.237, *p* < 0.01), family resilience (*r* = 0.436, *p* < 0.01), and negative coping (*r* = −0.234, *p* < 0.01). In the APIMeM, negative coping fully mediated the association between patients’ threat appraisal and their QoL, whereas caregivers’ threat appraisal was directly associated with poorer QoL. Family resilience from either dyad member predicted better patient QoL. Actor effects predominated, with patients’ threat appraisal predicting caregivers’ negative coping as a cross-dyad pathway.

**Conclusion:**

Acute leukemia patients and their primary family caregivers demonstrate divergent stress-coping pathways. For patients, mitigating maladaptive coping such as avoidance and resignation may protect QoL, whereas caregivers may benefit from interventions directly targeting threat perception and emotional burden. Family resilience shows a distinct pattern. It exerts protective effects on patient QoL but not on caregiver QoL, pointing to the need for tailored dyadic psychosocial care.

## 1 Introduction

Acute leukemia (AL) is a malignant clonal disorder of hematopoietic stem cells, characterized by rapid proliferation of abnormal leukemic cells in the bone marrow and infiltration into extramedullary tissues such as the liver, spleen, and lymph nodes, leading to suppression of normal hematopoiesis. Clinical manifestations include persistent fever, recurrent infections, progressive anemia, hemorrhage, and leukemic organ infiltration (Huang, 2009; Siegel et al., 2023). According to the American Cancer Society, approximately 6,550 new cases of acute lymphoblastic leukemia (ALL) and 20,800 new cases of acute myeloid leukemia (AML) were projected in the United States in 2024, with 1,330 and 11,220 deaths, respectively (American Cancer Society, 2024). In China, the incidence rate of AML was 1.62 per 100,000 population, compared with 0.69 per 100,000 for ALL; AML is more prevalent in adults (Ge and Xu, 2018). AL carries high morbidity and mortality, with relatively low survival, remission, and cure rates. Treatment is intensive and costly, imposing substantial economic burdens on families and healthcare systems (Rose-Inman and Kuehl, 2017; Bosshard et al., 2018). There is a pressing need for nursing research focused on AL patients to address these challenges.

The AL patients and their primary family caregivers are interdependent throughout the illness trajectory. The diagnosis and treatment of AL cause substantial psychological distress and reduced quality of life (QoL) in patients and also affect the physical and mental health of caregivers (Yucel et al., 2021; Wang et al., 2020; Yu et al., 2017; Liang et al., 2019). Beyond financial burdens and caregiving responsibilities, caregivers are repeatedly exposed to patients’ fears and anxieties over time (Wang et al., 2021; Tao et al., 2024; Xie et al., 2016). Caregivers themselves are therefore significantly affected by the challenges of AL.

The AL patients have been found to have significantly lower QoL than the norms reported by Cella, with QoL scores correlated with gender, occupation, and chemotherapy status (Yu et al., 2017; Wang et al., 2021). Primary family caregivers also face substantial burdens, including daily care responsibilities and psychological distress related to the potential loss of a loved one (Wang et al., 2021; Jibb et al., 2022), which compromises their mental and physical health and overall QoL. Most caregivers are parents or spouses, and their QoL is significantly lower than that of the general population (Yu et al., 2017). A significant negative correlation has been reported between patients’ social QoL and caregivers’ physical health-related QoL (Chen et al., 2023). The QoL of AL patients and their caregivers remain understudied and warrants further research and clinical attention.

According to the theory of stress and coping (Lazarus and Folkman, 1984), cognitive appraisal and coping are fundamental processes through which individuals evaluate stressful events and adapt to their implications. When facing a stressor, individuals engage in cognitive appraisal and select coping strategies before implementing adaptive responses, a process that influences subsequent psychosocial and physical outcomes (Folkman, 1997; Folkman et al., 1986). Cognitive appraisal refers to an individual’s evaluation of the severity and impact of AL, while coping involves mobilizing resources such as interpersonal and emotional support and implementing coping strategies. As a highly stressful life event, AL has adverse physical and psychological effects on both patients and caregivers. Disease recurrence has been associated with physical deterioration, negative illness perceptions, fear of progression, and uncertainty and helplessness (Gu et al., 2019; Koenigsmann et al., 2006; Lee et al., 2019). These psychological burdens often lead to passive coping strategies such as avoidance and resignation in dealing with treatment and daily care. Female cancer patients using maladaptive coping strategies such as denial, self-blame, and self-distraction have reported higher perceived stress and lower QoL (Yeh et al., 2023). Such maladaptive responses can exacerbate patient distress and intensify caregiver burden, further reducing patient QoL. Caregivers also often experience high perceived stress and are susceptible to anxiety and depression (Wang et al., 2021; Unsar et al., 2021; Hareendran et al., 2020), which adversely affects their well-being and QoL and underscores the reciprocal impact of patient and caregiver distress.

Family resilience refers to a family’s ability to acquire resources and strengths in the face of adversity, involving the mobilization of internal and external resources, negotiation among family members, and collective adaptation (McCubbin and Patterson, 1983; McCubbin et al., 1996). Highly resilient families typically show self-confidence, empathy, healthy relationships, and psychosocial maturity. Family resilience can serve as a protective factor by promoting psychological adjustment and enhancing QoL for both AL patients and their caregivers. Macía et al. (2020) found that resilience was significantly correlated with adaptive coping and positively associated with QoL in cancer patients, possibly because resilience mitigates negative emotions such as anxiety, depression, and stress, facilitating psychological adaptation to illness. Ke et al. (2023) found that family resilience was positively associated with QoL in both lung cancer patients and their caregivers, and higher family resilience was linked to more constructive dyadic coping strategies, which in turn improved QoL (Park et al., 2023). However, the mechanisms remain unclear, and no quantitative study has specifically examined the reciprocal relationship between family resilience and QoL among AL patient-caregiver dyads.

Cancer is a significant stressor affecting both patients and their primary family caregivers, and a dyadic approach is warranted across the treatment trajectory (Li et al., 2024; Chen et al., 2025). Previous studies have examined bivariate associations among cognitive appraisal, coping styles, family resilience, and QoL, yet the complex interrelationships and mechanisms linking these variables remain poorly understood. Few studies have tested these variables simultaneously within a dyadic framework, and research including both AL patients and their primary family caregivers is scarce. Two distinct but complementary theoretical frameworks inform this study. The Theory of Stress and Coping (Lazarus and Folkman, 1984) explain adaptation at the individual level: cognitive appraisal influences coping strategies, which in turn affect outcomes such as QoL. The Family Stress Theory (McCubbin and Patterson, 1983) focuses on adaptation at the family level, where resources such as family resilience mediate the impact of stressors on individual and family functioning. The Actor-Partner Interdependence Mediation Model (APIMeM) framework integrates these two levels, examining individual coping processes and family-level resource effects while capturing interpersonal influence within the dyad. This study therefore aims to identify factors associated with QoL in AL patient-caregiver dyads. Using APIMeM, the study tests actor effects (an individual’s cognitive appraisal influencing their own QoL) and partner effects (one member’s appraisal influencing the other’s QoL). Coping styles and family resilience are examined as potential mediators of these pathways.

## 2 Materials and methods

### 2.1 Study design

This descriptive cross-sectional survey was conducted from June 2022 to June 2023. The research used convenience sampling, and the participants were hospitalized patients with AL and their primary family caregivers that from five tertiary grade A hospitals in China.

### 2.2 Data collection and participants

In total, 336 patients and their primary family caregivers were recruited, and the investigation was completed during the period of hospitalization. After excluding those with missing answers, incomplete answers, and invalid or inaccurate data, 308 dyads questionnaires remained for analysis (resulting in an effective response rate of 91.67%). The inclusion criteria for patients were as follows:(1) had a diagnosis of myeloid neoplasms and AL based on the 2016 WHO classification (Arber et al., 2016); (2) were aged ≥18 years; (3) had clear consciousness, without mental disorders or communication barriers; (4) in need of family care in daily life, and (5) provided informed consent to participate voluntarily. Patients were excluded if they: (1) people who do not know their condition yet; (2) had cancer metastasis, were in critical condition or had other serious physical diseases; (3) lived alone or with a nanny or carer, or (4) had poor adherence. The inclusion criteria for primary family caregivers were as follows: (1) were aged ≥18 years old; (2) patient-appointed primary family caregivers who lives with the patient and cares for him/her for at least 4 h a day; (3) can make decisions about and take responsibility for a patient’s clinical care; (4) had clear consciousness, without mental disorder and communication barrier; (5) informed consent to participate voluntarily. Primary family caregivers were excluded if they: (1) suffered other stressful events recently and are not yet emotionally stable; (2) had other serious physical diseases; (3) had poor adherence.

### 2.3 Outcomes and measurements

#### 2.3.1 Sociodemographic characteristics

Demographic and clinical characteristics were collected from both patients and their primary family caregivers. The variables included sex, age, ethnicity, occupation, residential location, marital status, educational level, occupational status, per capita monthly household income, medical payment method, number of chemotherapies, access to sources of disease-related knowledge, primary family caregivers, presence of other chronic diseases, level of disease-related knowledge, level of patient self-managed, average daily care time.

#### 2.3.2 Quality of life (QoL) measurements

The Functional Assessment of Cancer Therapy-Leukemia (FACT-Leu) was used to evaluate the QoL of patients with AL over the preceding 7 days (Cella et al., 2012). The instrument comprises 44 items across five domains: physical well-being (PWB), functional well-being (FWB), emotional well-being (EWB), social/family well-being (SWB), and additional concerns. Responses were recorded on a 5-point Likert scale ranging from 0 (“not at all”) to 4 (“very much”). Total scores range from 0 to 176, with higher scores indicating better QoL. In the present study, the FACT-Leu demonstrated excellent internal consistency, with a Cronbach’s α of 0.935.

The 12-Item Short-Form Health Survey (SF-12) was employed as a generic instrument to assess the QoL of primary family caregivers over the preceding 4 weeks (Ware et al., 1996). The questionnaire comprises 12 items grouped into eight domains: physical functioning (PF), role physical (RP), bodily pain (BP), general health (GH), vitality (VT), social functioning (SF), role emotional (RE), and mental health (MH). Standardized scoring was applied, with both physical and mental component summary scores transformed to a range of 0 to 100. Higher scores indicate better perceived QoL. In this study, the SF-12 demonstrated high internal consistency, with a Cronbach’s *α* of 0.892.

#### 2.3.3 Cognitive appraisal of health scale

The Cognitive Appraisal of Health Scale (CAHS), originally developed by Kessler (1998), was utilized in its Chinese version as translated and culturally adapted by Lin et al. (2015). The CAHS comprises two components: primary appraisal and secondary appraisal. This study employed the primary appraisal section to examine how patients with AL and their primary family caregivers cognitively evaluate health-related stressful encounters. This subscale contains 23 items across four dimensions: threat, challenge, harm/loss, and benign/irrelevant. Items are rated on a 5-point Likert scale from 1 (“strongly disagree”) to 5 (“strongly agree”), with total scores ranging from 23 to 115, higher scores indicate stronger endorsement of primary appraisal. The primary appraisal subscale was selected for two main reasons. First, in Lazarus’s transactional model of stress and coping, primary appraisal (the judgment of an event’s nature) is the direct antecedent of coping strategies and represents the core appraisal process of interest in this study; secondary appraisal primarily concerns the judgment of one’s coping resources and efficacy. Second, the four dimensions of primary appraisal (threat, challenge, harm/loss, benign/irrelevant) capture the main aspects of individuals’ cognitive appraisal of illness events. Cronbach’s α for this subscale was 0.635 (patients) and 0.712 (caregivers). The patient α was slightly below the 0.70 threshold, likely because the primary appraisal subscale contains both positive (challenge, benign/irrelevant) and negative (threat, harm/loss) dimensions. McDonald’s ω, which does not assume equal factor loadings, was 0.829, and all four subscales had *α* > 0.70, indicating acceptable reliability.

#### 2.3.4 Coping style questionnaire

The Trait Coping Style Questionnaire (TCSQ), developed by Jiang and Zhu (1999), was used to assess the influence of stress on an individual’s psychosomatic health. This instrument consists of 20 items grouped into two dimensions: positive coping (PC) and negative coping (NC). Responses are recorded on a 5-point Likert scale ranging from 1 (“certainly not”) to 5 (“certainly”). Total scores range from 20 to 100, with higher scores on each subscale indicating a stronger tendency toward the corresponding coping style. In the original validation, Cronbach’s α was 0.69 for the PC subscale and 0.70 for the NC subscale. In the present study, Cronbach’s α values were 0.882 (PC) and 0.849 (NC) for patients, and 0.812 (PC) and 0.821 (NC) for primary family caregivers.

#### 2.3.5 Family hardiness index

The Family Hardiness Index (FHI), developed by McCubbin et al. (1996), was used to evaluate hardiness as a characteristic of family stress resistance and adaptive capacity. The Chinese version of the FHI was translated and culturally adapted by Liu et al. (2014). This scale comprises 20 items assessing three dimensions: commitment, challenge, and control. Items are rated on a 4-point Likert scale ranging from 1 (“strongly disagree”) to 4 (“strongly agree”). Total scores range from 20 to 80, with higher scores indicating greater family hardiness. The Cronbach’s α of the Chinese version was previously reported as 0.803. In this study, Cronbach’s *α* was 0.819 for patients and 0.838 for primary family caregivers.

### 2.4 Statistical analysis

All statistical analyses were performed using IBM SPSS Statistics 26.0 and R 4.6.0. Descriptive statistics were computed for sample characteristics; categorical variables are reported as frequencies and percentages, and continuous variables as means and standard deviations. Pearson correlations were used to examine the relationships among the study variables.

An APIMeM was fitted with the lavaan package (version 0.7.2) in R. The model tested direct and indirect associations of cognitive appraisal with QoL through coping styles and family resilience, estimating both actor effects (within-person) and partner effects (cross-partner). It comprised eight observed variables-cognitive appraisals, coping styles, family resilience, and QoL for each dyad member-with coping styles and family resilience specified as parallel mediators. All variables were standardized prior to analysis. Maximum likelihood estimation was used.

Specific, total indirect, and total effects were estimated for each actor and partner pathway. Indirect effects were evaluated with 5,000 bias-corrected and accelerated bootstrap resamples; a 95% confidence interval excluding zero indicated significance. Model fit was assessed using *χ*^2^/df, CFI, TLI, RMSEA, and SRMR. All tests were two-tailed, with *p* < 0.05 considered significant.

## 3 Results

### 3.1 Sample characteristics

The mean ages of patients AL and their primary family caregivers were 46.52 (SD = 15.22) and 45.81 (SD = 12.74) years, respectively. Among the patients, males constituted a higher proportion (54.87%, *n* = 169) compared to females (45.13%, *n* = 139). Most patients (58.12%, *n* = 179) resided in rural areas, while 41.88% (*n* = 129) lived in urban or town settings. Spouses were the most common type of primary family caregiver (50.65%, *n* = 156), followed by children (23.38%, *n* = 72), parents (21.10%, *n* = 65), and others (4.87%, *n* = 15) (see Table 1).

**Table 1:** Demographic and cancer-related characteristics (308 dyads).

Variable	Patients with AL *n* (%)	Primary family caregivers *n* (%)
Age		
18 ~ 39	119 (38.64)	113 (36.69)
40 ~ 59	123 (39.94)	151 (49.03)
60 ~ 80	66 (21.42)	44 (14.28)
Gender		
Male	169 (54.87)	134 (43.51)
Female	139 (45.13)	174 (56.49)
Ethnicity		
Han	304 (98.70)	304 (98.70)
Other ethnic minorities	4 (1.30)	4 (1.30)
Residential location		
Rural	179 (58.12)	179 (58.12)
Urban or town settings	129 (41.88)	129 (41.88)
Marital status		
Not married	44 (14.29)	25 (8.12)
Married	253 (82.14)	277 (89.94)
Divorced	7 (2.27)	3 (0.97)
Widowed	4 (1.30)	3 (0.97)
Educational level		
Primary school or below	52 (16.88)	49 (15.91)
Lower secondary or technical secondary school	121 (39.29)	123 (39.94)
High school or junior college	87 (28.25)	93 (30.19)
Bachelor’s degree and above	48 (15.58)	43 (13.96)
Occupational status		
Workers	28 (9.09)	42 (13.64)
Farmers	126 (40.91)	143 (46.43)
Cadre or staff	33 (10.71)	32 (10.39)
Self-employed	10 (3.25)	18 (5.84)
Unemployed	53 (17.21)	32 (10.39)
Retirees	23 (7.47)	20 (6.49)
Others	35 (11.36)	21 (6.82)
Access to sources of disease-related knowledge		
Medical workers	205 (66.56)	206 (66.88)
Internet	37 (12.01)	23 (7.47)
Others	66 (21.43)	79 (25.65)
Level of disease-related knowledge		
Very well informed	40 (12.99)	39 (12.66)
Fairly well informed	38 (12.34)	51 (16.56)
Have some understanding	216 (70.13)	211 (68.51)
Do not know anything about it	14 (4.54)	7 (2.27)
Per capita monthly household income		
<1,000	85 (27.60)	–
1,000 ~ 3,000	105 (34.09)	–
3,001 ~ 5,000	88 (28.57)	–
5,001 ~ 10,000	27 (8.77)	–
>10,000	6 (0.97)	–
Medical payment method		
Urban residents’ medical insurance	44 (14.29)	–
Rural cooperative medical care	160 (51.95)	–
Commercial medical insurance	2 (0.65)	–
Urban workers’ medical insurance	68 (22.08)	–
Provincial and municipal health insurance	24 (7.79)	–
Fully self-financed	8 (2.59)	–
Others	2 (0.65)	–
Number of chemotherapies (time)		
0 time	59 (19.16)	–
1 ~ 3 times	107 (34.74)	–
4 ~ 6 times	69 (22.40)	–
7 ~ 9 times	26 (8.44)	–
>9 times	47 (15.26)	–
Primary family caregivers		
Parents	–	65 (21.10)
Spouses	–	156 (50.65)
Children	–	72 (23.38)
Others	–	15 (4.87)
Presence of other chronic diseases		
Yes	–	52 (16.88)
No	–	256 (83.12)
Level of patient self-managed		
Fully self-managed	–	55 (17.86)
Basically self-managed	–	141 (45.78)
Partly self-managed	–	106 (34.42)
Completely incapable of self-managed	–	6 (1.94)
Average daily care time		
4 ~ 8 times	–	54 (17.53)
9 ~ 12 times	–	75 (24.35)
>12 times	–	179 (58.12)

“–” means that the information is the same for both parties or is specific to the individual.

### 3.2 Correlation analysis

Table 2 displays the bivariate correlations among QoL, cognitive appraisal, positive coping, negative coping, and family resilience in patients with AL and their primary family caregivers. The QoL of AL patients showed significant positive correlations with caregivers’ QoL (*r* = 0.237, *p* < 0.01), positive coping (*r* = 0.242, *p* < 0.01), and family resilience (*r* = 0.436, *p* < 0.01). Furthermore, a significant negative correlation was identified between patients’ QoL and caregivers’ negative coping (*r* = −0.234, *p* < 0.01).

**Table 2:** Descriptive statistics and correlations among the variables (308 dyads).

Variables	*M* ± SD	1	2	3	4	5	6	7	8	9	10
1. Quality of life (PAL)	55.15 ± 15.98	1	—	—	—	—	—	—	—	—	—
2. Cognitive appraisal (PAL)	70.97 ± 7.73	−0.198^**^	1	—	—	—	—	—	—	—	—
3. Positive coping (PAL)	31.66 ± 7.47	0.523^**^	0.107	1	—	—	—	—	—	—	—
4. Negative coping (PAL)	30.18 ± 6.57	−0.466^**^	0.463^**^	−0.341^**^	1	—	—	—	—	—	—
5. Family resilience (PAL)	56.10 ± 7.00	0.489^**^	−0.100	0.586^**^	−0.424^**^	1	—	—	—	—	—
6. Quality of life (PFC)	68.91 ± 18.05	0.237^**^	−0.067	0.082	−0.199^**^	0.098	1	—	—	—	—
7. Cognitive appraisal (PFC)	66.05 ± 8.61	−0.006	0.211^**^	0.018	0.173^**^	0.015	−0.258^**^	1	—	—	—
8. Positive coping (PFC)	32.59 ± 7.13	0.242^**^	0.049	0.349^**^	−0.139^*^	0.283^**^	0.206^**^	−0.015	1	—	—
9. Negative coping (PFC)	30.61 ± 6.74	−0.234^**^	0.209^**^	−0.137^*^	0.357^**^	−0.279^**^	−0.259^**^	0.257^**^	−0.142^*^	1	—
10. Family resilience (PFC)	56.26 ± 6.74	0.436^**^	−0.056	0.452^**^	−0.341^**^	0.597^**^	0.234^**^	−0.084	0.522^**^	−0.431^**^	1

PAL, patients with acute leukemia; PFC, primary family varegivers; ^*^p < 0.05, ^**^p < 0.01.

Multiple linear regression was conducted to identify predictors of QoL among AL patients and their primary family caregivers. QoL was the dependent variable. Independent variables included socio-demographic characteristics that differed significantly by QoL, cognitive appraisal, positive coping, negative coping, and family resilience. In the patient model, significant predictors were occupation status, cognitive appraisal, positive coping, negative coping, and family resilience. In the primary family caregiver model, significant predictors were the primary family caregivers, level of patient self-managed, cognitive appraisal, and positive coping.

### 3.3 Mediation analysis with APIMeM

An APIMeM was specified to examine the dyadic associations among cognitive appraisal, coping, family resilience, and QoL, with coping and family resilience as parallel mediators. Positive and negative coping were examined in separate models. In both models, the paths from cognitive appraisal to family resilience were non-significant (all *p* > 0.13); family resilience was therefore re-specified as an exogenous direct predictor of QoL rather than a mediator. Because the indirect effects through negative coping were stronger and more consistent, the negative-coping model is reported below as the primary analysis; the positive-coping model is summarized as a supplementary analysis. Covariates included patient occupation status, primary family caregivers, and level of patient self-managed. All continuous variables were standardized; maximum likelihood estimation with 5,000 BCa bootstrap resamples was used (lavaan 0.7–2, R 4.6.0).

Model fit was: *χ*^2^/df = 2.30; CFI = 0.948; TLI = 0.825; RMSEA = 0.065; SRMR = 0.043. Overall, these indices indicated acceptable fit to the data, although TLI fell below the conventional 0.90 threshold. The model explained 40.9 and 20.7% of variance in patient and caregiver QoL, and 23.4 and 12.5% in patient and caregiver negative coping (Table 3 and Figure 1).

**Table 3:** Standardized path coefficients, indirect effects, and model fit indices for the actor–partner interdependence mediation model (*N* = 308 dyads).

Path	*β*	95% BCa CI	*P*
a paths: Cognitive appraisal → negative coping			
Patient CA → patient NC (actor)	0.418***	[0.311, 0.498]	<0.001
Caregiver CA → patient NC (partner)	0.081	[−0.015, 0.167]	0.087
Patient CA → caregiver NC (partner)	0.139*	[0.034, 0.247]	0.012
Caregiver CA → caregiver NC (actor)	0.195**	[0.056, 0.346]	0.009
b paths and direct effects → patient QoL			
Patient NC → patient QoL	−0.244***	[−0.370, −0.113]	<0.001
Caregiver NC → patient QoL	0.032	[−0.074, 0.132]	0.546
Patient FR → patient QoL	0.251***	[0.120, 0.379]	<0.001
Caregiver FR → patient QoL	0.203**	[0.078, 0.311]	0.001
Patient CA → patient QoL (direct)	−0.043	[−0.172, 0.090]	0.538
b paths and direct effects → caregiver QoL			
Patient NC → caregiver QoL	−0.080	[−0.224, 0.050]	0.243
Caregiver NC → caregiver QoL	−0.064	[−0.195, 0.062]	0.327
Patient FR → caregiver QoL	−0.049	[−0.186, 0.093]	0.504
Caregiver FR → caregiver QoL	0.171*	[0.026, 0.313]	0.021
Caregiver CA → caregiver QoL (direct)	−0.231***	[−0.347, −0.109]	<0.001
Indirect effects on patient QoL			
CA → patient NC → QoL (actor)	−0.102**	[−0.163, −0.044]	0.001
CA → caregiver NC → QoL (partner)	0.004	[−0.009, 0.021]	0.579
Total indirect effect	−0.097**	[−0.158, −0.040]	0.001
Total effect	−0.140*	[−0.262, −0.015]	0.033
Indirect effects on caregiver QoL			
CA → caregiver NC → QoL (actor)	−0.012	[−0.053, 0.011]	0.363
CA → patient NC → QoL (partner)	−0.007	[−0.031, 0.003]	0.383
Total indirect effect	−0.019	[−0.082, 0.004]	0.211
Total effect	−0.250***	[−0.369, −0.133]	<0.001
Cross-dyad indirect effects			
Caregiver CA → patient NC → patient QoL	−0.020	[−0.053, −0.001]	0.125
Caregiver CA → caregiver NC → patient QoL	0.006	[−0.014, 0.038]	0.583
Caregiver CA → patient QoL (total cross)	−0.013	[−0.089, 0.031]	0.375
Patient CA → patient NC → caregiver QoL	−0.034	[−0.096, 0.018]	0.254
Patient CA → caregiver NC → caregiver QoL	−0.009	[−0.035, 0.006]	0.413
Patient CA → caregiver QoL (total cross)	−0.043	[−0.103, 0.023]	0.129

β = standardized path coefficient; BCa = bias-corrected and accelerated bootstrap (5,000 resamples); CI = confidence interval; CA = cognitive appraisal; NC = negative coping; PC = positive coping; FR = family resilience; QoL = quality of life. *p < 0.05, **p < 0.01, ***p < 0.001.

**Figure 1:**
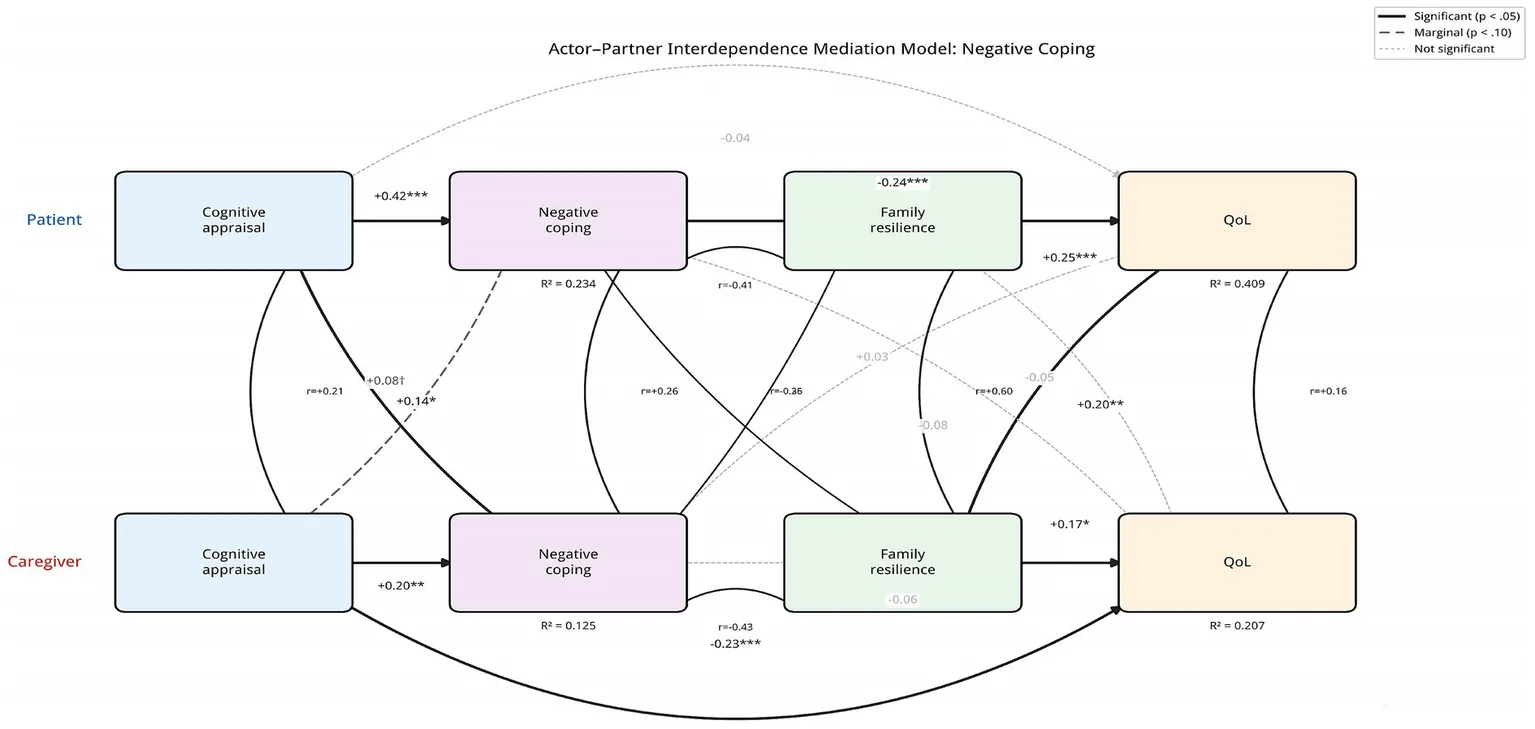
Path diagram of the Actor-Partner Interdependence Mediation Model (APIMeM). *p < 0.05. **p <0.01. ***p < 0.001.

Patients’ cognitive appraisal predicted their own negative coping (*β* = 0.42, 95% CI [0.31, 0.50], *p* < 0.001) and that of their caregivers (*β* = 0.14, 95% CI [0.03, 0.25], *p* = 0.012). Caregivers’ appraisal likewise predicted their own negative coping (*β* = 0.20, 95% CI [0.06, 0.35], *p* = 0.009), but the crossover path to patients’ coping was not significant (*β* = 0.08, *p* = 0.087).

Greater negative coping predicted poorer QoL among patients (*β* = −0.24, 95% CI [−0.37, −0.11], *p* < 0.001); neither members’ coping predicted the other’s QoL (both *p* > 0.24). Family resilience predicted better QoL for both members: patient resilience predicted patient QoL (*β* = 0.25, *p* < 0.001), caregiver resilience predicted patient QoL (*β* = 0.20, *p* = 0.001) and caregiver QoL (*β* = 0.17, *p* = 0.021). Patients’ cognitive appraisal showed no direct effect on their own QoL in the full model (*β* = −0.04, *p* = 0.538), whereas caregivers’ appraisal retained a direct effect on theirs (*β* = −0.23, 95% CI [−0.35, −0.11], *p* < 0.001).

The actor indirect effect for patients was significant: cognitive appraisal predicted poorer QoL through greater negative coping (*β* = −0.10, 95% BCa CI [−0.16, −0.04], *p* = 0.001). The partner-mediated path through caregivers’ coping was not significant (*β* = 0.004, *p* = 0.579). The total indirect effect (*β* = −0.10, *p* = 0.001) and total effect (*β* = −0.14, 95% CI [−0.26, −0.02], *p* = 0.033) were both significant for patients. For caregivers, neither the actor (*β* = −0.01, *p* = 0.363) nor partner (*β* = −0.01, *p* = 0.383) indirect effect reached significance, and the total indirect effect was null (*β* = −0.02, *p* = 0.211), although the total effect on caregiver QoL was significant (*β* = −0.25, 95% CI [−0.37, −0.13], *p* < 0.001). No cross-dyad indirect effect was significant (all *p* > 0.12).

A parallel model with positive coping yielded a significant actor indirect effect for patients (*β* = 0.07, 95% BCa CI [0.02, 0.12], *p* = 0.005), though the direct path remained significant (*β* = −0.20, *p* = 0.002). No partner indirect effects were significant. Only the direct effect was significant for caregivers (*β* = −0.26, *p* < 0.001). The indirect effect through negative coping (|*β*| = 0.10) was larger in magnitude than that through positive coping (*β* = 0.07).

An exploratory model substituted the 13-item threat/harm-loss subscale (*α* = 0.89/0.91) for the total CAHS (*α* = 0.64/0.71). Both crossover paths to negative coping were significant (patient→ caregiver: *β* = 0.19, *p* = 0.001; caregiver → patient: *β* = 0.15, *p* = 0.003), and actor paths were stronger (*β* = 0.53 and 0.27, both *p* < 0.001). The indirect effect was not significant, however, because threat/harm appraisal correlated strongly with QoL (*r* = −0.66), leaving little unique variance attributable to coping. This model fit poorly (CFI = 0.85, RMSEA = 0.22) and is best regarded as preliminary, though the significant crossover paths corroborate the primary analysis.

## 4 Discussion

Grounded in stress and coping theory and family resilience theory, this study used APIMeM to examine the links among cognitive appraisal, coping styles, family resilience, and QoL in patient-primary family caregiver dyads in AL. For patients, negative coping fully mediated the association between threat appraisal and poorer QoL, whereas positive coping produced a smaller partial indirect effect. For primary family caregivers, threat appraisal was directly linked to poorer QoL, and neither coping nor family resilience mediated this association. Family resilience, whether reported by patients or primary family caregivers, independently predicted better patient QoL. Actor effects predominated. The one significant cross-dyad path ran from patients’ threat appraisal to caregivers’ negative coping. Patients and primary family caregivers within the same dyad therefore appear to follow distinct stress-coping pathways, with different implications for dyad-focused psychosocial care.

Negative coping fully mediated the association between patients’ threat appraisal and QoL. Once coping entered the model, the direct path from appraisal to QoL was no longer significant. For patients with AL, the diagnosis often arrives abruptly, chemotherapy is intensive, hospitalizations are frequent, and prognosis remains uncertain. Threat appraisal in this context is not an abstract construct but a lived daily experience. When patients perceive the illness as exceeding their coping capacity, they tend toward avoidance and resignation—avoiding discussions about prognosis with clinicians, concealing distress from family, or adopting a fatalistic outlook. These responses provide immediate relief, but they also accrue costs: treatment decisions are made passively, emotional distress remains unprocessed across successive chemotherapy cycles, and available family and social support goes untapped. As this pattern of withdrawal persists over the course of illness, QoL declines. In AL, where threat perception is likely widespread, patients with better QoL are not necessarily those who perceive less threat but those who rely less on avoidance. Positive coping, by contrast, accounted for substantially less variance. Cognitive reframing and active engagement require cognitive and emotional reserves that AL patients often lack-reserves shaped not only by individual temperament but also by the social support and material resources available to them. Prognostic uncertainty and the physical burden of treatment deplete these reserves, and the protective effect of positive coping weakens accordingly. The finding that the adverse effect of negative coping exceeds the protective effect of positive coping is consistent with the well—documented negativity bias: negative events capture attention and memory more readily than positive ones, and this asymmetry amplifies under threat (Baumeister et al., 2001). For hematology nursing practice, the implication is clear. Routine assessment of avoidance and resignation, coupled with targeted interventions to reduce these behaviors, may benefit patients’ QoL more than interventions focused exclusively on promoting positive coping.

Primary family caregivers showed a distinct pattern. Their threat appraisal was directly associated with poorer QoL. The impact of a stressor on well-being is shaped by cognitive appraisal, and positive appraisal has been theorized as a protective factor for family resilience (Bailey et al., 2013). Patients and caregivers, however, follow distinct adaptation pathways. For patients, the illness threatens the body directly, and coping mediates the link between appraisal and QoL. For caregivers, the demands extend beyond physical care to household responsibilities, emotional labor, and persistent uncertainty about treatment outcomes. Patients can at least address their threat through personal coping efforts; primary family caregivers, by contrast, confront much that lies beyond their direct control-the diagnosis, the treatment regimen, its side effects. This lack of control may weaken the mediating role of coping, because caregivers have limited room to influence the illness trajectory through their own actions. Threat appraisal thus affects caregiver QoL more directly, without coping serving as a significant intermediary. This pattern carries concrete implications for hematology nursing. In the present sample, primary family caregivers were predominantly women (64.6%), most commonly spouses and parents, and many shouldered caregiving duties alongside ongoing work and family obligations. During induction chemotherapy, primary family caregivers are typically present at the bedside daily, managing treatment—related symptoms, coordinating medical information, and navigating the complexities of an unfamiliar hospital system. Their threat appraisal is embedded in these concrete, repeated experiences. Nursing interventions should therefore prioritize the direct assessment and alleviation of caregiver burden-through clear information provision, care coordination, and timely referral to medical social work, psychological counseling, and peer support resources when needed—rather than relying on coping-skills training as the primary approach. In the Chinese context, where family members customarily assume the bulk of caregiving with limited formal support and few institutional alternatives, this direct pathway from threat appraisal to impaired QoL may be especially pronounced.

Both patient and their primary family caregiver-reported family resilience independently predicted better patient QoL. Patient-reported resilience showed the stronger association. Cognitive appraisal was not associated with family resilience in either dyad member. This finding supports the view that family resilience is an enduring resource of the family system rather than a product of immediate cognitive appraisal. As Yu et al. (2018) note, family resilience reflects the capacity of a family to mobilize its resources and adapt under stress. In this study, most primary family caregivers held a relatively positive view of the illness, describing it as a challenge or benign event rather than a threat. For AL patients, families with greater resilience may communicate more openly about the disease, distribute caregiving tasks more effectively, and stay emotionally connected during hospitalization. Together, these qualities help sustain patients’ QoL. The benefits of family resilience were not shared equally, however. Family resilience predicted patient QoL significantly, but it did not predict caregiver QoL. Patients receive family care. During chemotherapy, primary family caregivers help with daily needs at the bedside, manage practical arrangements, and provide emotional reassurance. Primary family caregivers are the ones who provide that care. A family that functions well may place heavier expectations on its primary family caregiver, requiring greater coordination of family affairs, more consistent emotional support, and longer presence at the hospital. These added demands can cancel out the psychological benefit that family resilience might otherwise bring. Caregiving is ongoing and the disease course unpredictable, both strain caregivers’ psychological reserves. When primary family caregivers become overwhelmed by daily burdens, their own QoL suffers, and this may further weaken the patient’s psychological resilience and the family’s ability to adapt (Song et al., 2025).

Actor effects predominated, yet partner effects were also meaningful. Each member’s QoL was predicted primarily by their own appraisal and coping; at the same time, several cross-dyad pathways reached significance. Patients’ threat appraisal predicted caregivers’ negative coping. Patients’ and caregiver’ negative coping were significantly correlated. Caregiver-reported family resilience also predicted patient QoL. These findings point to genuine emotional and behavioral transfer within the dyad. In Chinese families, patients and primary caregivers form an interdependent unit bound by family responsibility and shared experience of serious illness (Cai et al., 2025; Lyons and Lee, 2018), and affective contagion, or the automatic spread of mood between close relationship partners, is well established (Neumann and Strack, 2000; Liu et al., 2025). Caregivers often try to remain composed at the bedside, but anxiety can surface through facial expression, tone of voice, or fatigue, and patients may pick up on these nonverbal cues. A patient’s fear or distress can weigh heavily on the caregiver, particularly when the caregiver feels powerless to change the situation. Mutual empathy can intensify this exchange. Partner effects were smaller than actor effects, perhaps because in the acute phase, psychological resources are directed toward one’s own appraisal and coping. These cross-dyad pathways, though modest, remain relevant to nursing practice. When assessing a patient’s psychological state, nurses should also observe the caregiver’s emotional responses, and vice versa. The significant association between patients’ and caregivers’ negative coping suggests that interventions targeting one member’s coping may have spillover effects on the other. Future longitudinal research should examine how these dyadic connections evolve over time, particularly as patients transition from acute treatment to remission and maintenance therapy.

### 4.1 Limitations

Several limitations should be acknowledged. First, the cross-sectional design precludes causal inference. The directionality of the observed relationships therefore cannot be established. Longitudinal or repeated-measures designs are needed to confirm temporal ordering and validate the current findings. Second, CFI and SRMR indicated acceptable fit, but TLI fell below 0.90. The model may therefore benefit from further specification. Fit indices should be interpreted with caution, and the proposed model requires replication in independent samples. Third, Cronbach’s α for the patient CAHS was 0.635, below the conventional 0.70 threshold. This may partly reflect the multidimensional structure of primary appraisal. McDonald’s ω was 0.83 and subscale reliabilities were acceptable. These figures point to adequate overall reliability. Even so, residual measurement error remains possible and may have attenuated some path coefficients. Finally, convenience sampling from five tertiary hospitals in a single city may limit the generalizability of the findings. Multi-center studies across diverse regions would strengthen sample representativeness.

## 5 Conclusion

This study employed the APIMeM framework to examine associations among cognitive appraisal, coping styles, family resilience, and QoL in AL patient and their primary family caregiver dyads. Negative coping fully mediated the link between patients’ threat appraisal and their QoL. Caregivers’ threat appraisal, by contrast, was directly associated with poorer QoL. Family resilience reported by either dyad member predicted better patient QoL but showed no significant association with caregiver QoL. Actor effects were dominant across pathways. Patients’ threat appraisal also significantly predicted caregivers’ negative coping, indicating cross-dyad influence. The distinct stress-coping patterns of patients and caregivers point to the value of dyadic psychosocial care.

## 6 Relevance for clinical practice

These findings point to several practical implications for clinical care. At diagnosis and again during intensive chemotherapy, nurses should conduct brief screenings for threat appraisal and negative coping in both patients and caregivers. For patients, the priority is reducing avoidance and resignation. Nurses can use motivational interviewing to help patients recognize avoidance patterns, offer guided emotional disclosure, and refer those in need to cognitive behavioral therapy (CBT) or acceptance and commitment therapy (ACT) (Finset, 2022; Fishbein and Arch, 2022; Huey et al., 2023). For primary family caregivers, threat perception warrants direct attention. Timely treatment information, clear expectations about the disease course, and validation of emotional responses are all important. Peer support groups and mindfulness-based programs can reduce anxiety, and referral to medical social workers can help connect families with financial assistance and practical support resources. At the family level, dyadic interventions should foster open communication about the illness. Evidence-based programs such as the FOCUS Program (Northouse et al., 2005) and DPST-HCT (Dyadic Problem-Solving Therapy for Hematopoietic Cell Transplantation; Posluszny et al., 2018) provide structured frameworks for promoting family communication, collaborative coping, and shared meaning. Interventions should also involve other family members in care and support and routinely assess caregiver burden. In clinical practice, nurses can take advantage of natural separations, such as when the patient is away for tests or the caregiver steps out briefly, to speak with each member individually, as family members may be reluctant to express distress in front of each other. Interventions initiated soon after diagnosis and during intensive treatment may be particularly beneficial.

## Data Availability

The raw data supporting the conclusions of this article will be made available by the authors, without undue reservation.
